# The *Legionella pneumophila* effector Lpg1137 is a homologue of mitochondrial SLC25 carrier proteins, not of known serine proteases

**DOI:** 10.7717/peerj.3849

**Published:** 2017-09-26

**Authors:** Marcin Gradowski, Krzysztof Pawłowski

**Affiliations:** 1 Department of Experimental Design and Bioinformatics, Faculty of Agriculture and Biology, Warsaw University of Life Sciences, Warszawa, Poland; 2 Department of Translational Medicine, Clinical Sciences, Lund University, Lund, Sweden

**Keywords:** Structure prediction, Bacterial effectors, Proteases, Mitochondrial carriers

## Abstract

Many bacterial effector proteins that are delivered to host cells during infection are enzymes targeting host cell signalling. Recently, *Legionella pneumophila* effector Lpg1137 was experimentally characterised as a serine protease that cleaves human syntaxin 17. We present strong bioinformatic evidence that Lpg1137 is a homologue of mitochondrial carrier proteins and is not related to known serine proteases. We also discuss how this finding can be reconciled with the apparently contradictory experimental results.

## Introduction

*Legionella pneumophila* is an intracellular pathogen that causes a deadly respiratory infection called Legionnaire’s disease. It typically infects amoebae, but can also enter human alveolar macrophages and proliferate within so-called Legionella-containing vacuoles (LCV) that are derived from the endoplasmic reticulum (Eisenreich & Heuner, 2016). To evade cellular defenses, for example to prevent the fusion of LCV with lysozymes, *L. pneumophila* and related species produce large repertoires of effectors that rewire host cell signalling (Burstein et al., 2016; Isaac & Isberg, 2014). A typical *L. pneumophila* strain produces approximately 300 different effectors that target processes as diverse as transcription and translation (Rolando & Buchrieser, 2014), and lipid (Viner et al., 2012), ubiquitin (Zhou & Zhu, 2015) and kinase signalling (Haenssler & Isberg, 2011). Many, if not the majority of bacterial effectors are distant homologues (Alto & Orth, 2012) or mimics of eukaryotic proteins (Shi et al., 2016).

The majority of *Legionella* effectors are experimentally uncharacterised, and a large fraction also remain unannotated despite large-scale bioinformatic endeavours. Such uncharacterised proteins evading function and structure prediction by automated bioinformatic pipelines can still be in many cases characterised *in silico* by careful application of diverse computational methods (Pawlowski, 2008). Effectors often turn out to be remote homologues of eukaryotic proteins, some harbouring well-known signalling domains, such as kinases (Dong et al., 2016) or proteases (Liu et al., 2017). Among the *Legionella* enzyme effectors, there are many cysteine proteases and metalloproteases, but very few serine proteases to date (Burstein et al., 2016).

Human Syntaxin 17 (Stx17) is a SNARE (soluble N-ethylmaleimide-sensitive factor attachment protein (SNAP) receptor) that localizes to endoplasmic reticulum (ER)-mitochondria contact sites. It performs diverse functions such as promoting mitochondrial fission and regulating ER Ca^2+^ homeostasis (Arasaki et al., 2015). Recently, it was reported that Stx17 is cleaved upon *L. pneumophila* infection, and that the cleavage depends on the presence of one of the multitude of as yet uncharacterised *L. pneumophila* effectors, Lpg1137(Arasaki et al., 2017). Further, this event “shuts down” communication between the ER and mitochondria.

Here, after an in-depth bioinformatic investigation, and unexpectedly for us, we can present strong bioinformatic evidence that Lpg1137 is actually a homologue of mitochondrial carrier proteins and is not related to known serine proteases.

## Methods

In order to explore possible distant sequence similarities of Lpg1137 to proteins of known structures, three established structural bioinformatic tools were used: FFAS03 (Jaroszewski et al., 2011), HHpred (Hildebrand et al., 2009) and Phyre2 (Bennett-Lovsey et al., 2008) with standard parameters and significance thresholds. The Phyre2 server was also used to build the three-dimensional structure model that was later visualized using Chimera software (Yang et al., 2012).

The multiple sequence alignment of mitochondrial carriers (MCs) and Lpg1137 homologues was built using the Muscle program (Edgar, 2004), and the sequence logos were created using the WebLogo server(Crooks et al., 2004).

For visual clustering of sequences, the CLANS algorithm (Frickey & Lupas, 2004) was applied to a set of representative sequences of MC pseudorepeats. The set was obtained by submitting three aligned MC pseudorepeats from the lncP protein (Refseq ID: WP_02722450, motifs defined by the Pfam database family Mito_carr, PF00153) (Finn et al., 2016) to two iterations of Jackhmmer search on the Uniprot database and by clustering with CD-HIT at a 35% sequence identity threshold (Huang et al., 2010). Then, the set was augmented by a set of homologues of Lpg1137 obtained from a Jackhmmer search (Finn, Clements & Eddy, 2011). CLANS was run with standard parameters using BLOSUM45 substitution matrix. For the graph, similarity relations with BLAST HSPs up to *E*-values of 1 were considered in order to visualize even distant similarities.

## Results and Discussion

**Figure 1:**
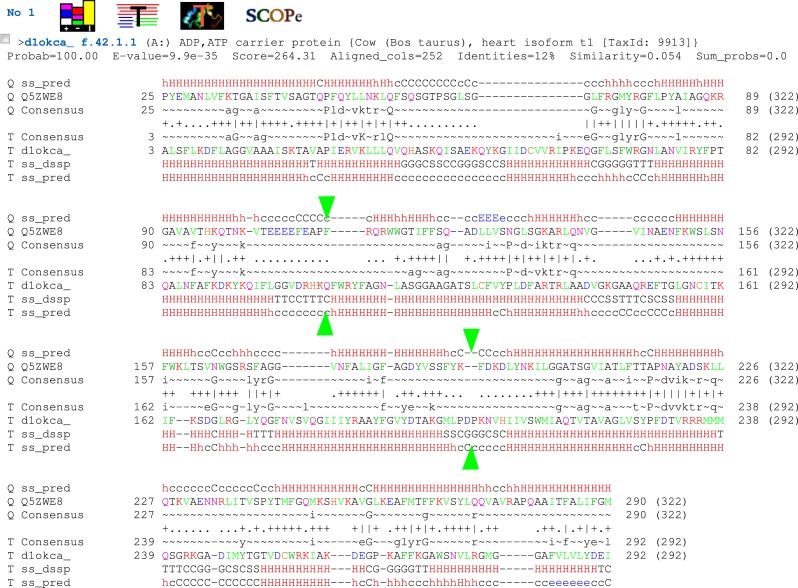
Protein sequence alignment between Lpg1137 (top, marked as Q5ZWE8) and bovine ADP, ATP carrier protein (bottom, marked as d1okca) obtained from the HHpred server. Predicted (ss_pred) and actual (ss_dssp) secondary structures shown: H-alpha helix, E-extended (beta strand), C-coil. Green triangles denote approximate borders of the three sequence repeats.

**Figure 2:**
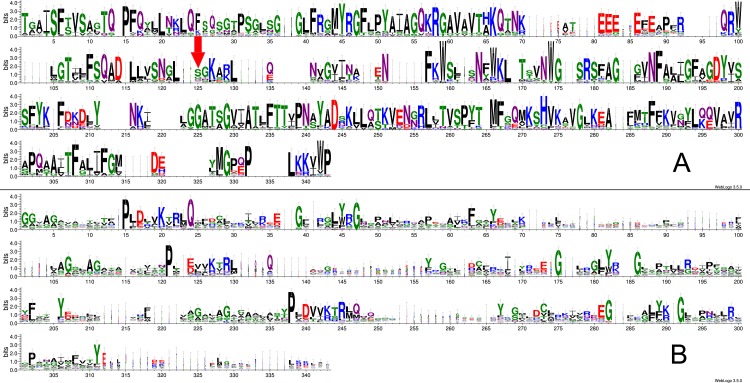
Sequence logos created from a combined Muscle alignment for Lpg1137 homologues (A) and human SLC mitochondrial carriers (B). Ser68 in Lpg1137 is located in column 125 of the sequence logo (red arrow). The Weblogo server was used (Crooks et al., 2004). The following residues are also shown in the structure model (Fig. 3): P15, G47, G175, P239 (numbering indicating positions in the sequence logo).

Initially, the report by Arasaki and co-workers (Arasaki et al., 2017) of a novel effector serine protease prompted us to undertake sequence exploration with the expectation of finding more similar effector proteases. In a recent bioinformatic exploration of *Legionella* effectors, Lpg1137 homologues were found in 16 out of 41 species studied, making it a relatively widespread effector (Burstein et al., 2016). Although a Blast sequence search did not yield any obvious Lpg1137 homologues outside the *Legionella* and *Fluoribacter* genera, to our surprise, three independent bioinformatic tools for remote sequence similarity recognition (FFAS03, Phyre2, HHpred) indicated statistically significant similarity of Lpg1137 to mitochondrial carrier proteins (MCs, also known in mammals as solute carrier family 25, SLC25; see Table 1). The broad region of sequence similarity between Lpg1137 and the carrier proteins suggests it is likely that Lpg1137 forms a standard MC structure with a pseudo-threefold symmetry with six transmembrane helices (Nury et al., 2006; Pebay-Peyroula et al., 2003). The three sequence repeats, albeit not obvious to the eye, are visible upon inspection of an HHpred alignment to a MC structure (see Fig. 1). Sequence logos of the repeats in homologues of Lpg1137, compared to sequence logo of the eukaryotic mitochondrial carriers (See Figs. 2A, 2B respectively) support the structural similarity by highlighting the conservation of structurally important Pro and Gly residues (e.g., Pro at positions 15 and 239 in the logos or the YxG motif at positions 45–47). These prolines and glycines are among the most conserved residues among the MC proteins (Wohlrab, 2005). Since the logos were created from an unbiased common sequence alignment of the Lpg1137 homologues and the eukaryotic mitochondrial carriers, the conservation of these residues is noteworthy.

**Table 1:** Top structure predictions for Lpg1137.

Bioinformatic tool for structure prediction	Top hit: PDB code, name	Statistical significance for top hit	Region of Lpg1137 aligned to the hit	Sequence identity in the alignment
FFAS03	2lck, Mitochondrial uncoupling protein 2 [Mus musculus]	*Z*-score = − 45.6	32 − 294	11%
HHpred	1okc, ADP, ATP carrier protein [Bos taurus]	*E*-value =9.9e − 35	25 − 290	12%
Phyre2	4c9q, mitochondrial adp/atp carrier isoform 32 [yeast]	Confidence =89.3	33 − 294	14%

The finding of Lpg1137 similarity to mitochondrial carriers raises the obvious question: how can this be rationalized, given the convincing experimental data by Arasaki et al.? Actually, what these authors have shown is that the presence of the *L. pneumophila* Lpg1137 protein in transfected HeLa cells resulted in the cleavage of host syntaxin 17 (Stx17). They have also demonstrated that this cleavage is not observed when Ser68 of Lpg1137 (hypothesized to be the catalytic residue) is mutated to alanine or when a serine protease inhibitor is applied. However, the following scenario could be at play. Lpg1137, likely located in the mitochondrial inner membrane as reported by Arasaki et al. may activate an undisclosed serine protease, either directly, e.g., by physical interaction, or indirectly, e.g., by providing a required concentration of a small molecule it may be transporting, e.g., ATP. Allosteric activation of proteases is a known mechanism, described for cysteine proteases and serine proteases alike, and it may involve binding small molecules, dimerization, or binding of an accessory protein (Arutyunova et al., 2014; De Regt et al., 2015; Lupardus et al., 2008; Zuhlsdorf et al., 2015). Such a mode of activation would explain the dependence of Stx17 cleavage on the presence of Lpg1137 and on serine protease inhibitors. Alternatively, interaction with Lpg1137 may make syntaxin 17 prone to cleavage. The cleavage might be executed by an endogenous host protease or by an effector protease. However, experiments, not bioinformatic predictions, should provide the definitive answer as to the functional identity of the Lpg1137 proteins.

Assuming the mitochondrial carrier prediction is correct, our bioinformatic analysis does not allow at this stage the prediction of detailed molecular function for Lpg1137. The known mitochondrial carriers exhibit great functional variability by transporting many diverse solutes (Palmieri & Monne, 2016). The sequence features of Lpg1137 and close homologs in the functionally important regions, e.g., the MC selectivity filter (Nury et al., 2006), do not permit it to be ascribed with confidence to any of the carrier subfamilies. Also, we cannot exclude that Lpg1137 might be a “pseudo-carrier”, a carrier-like molecule lacking carrier activity.

**Figure 3:**
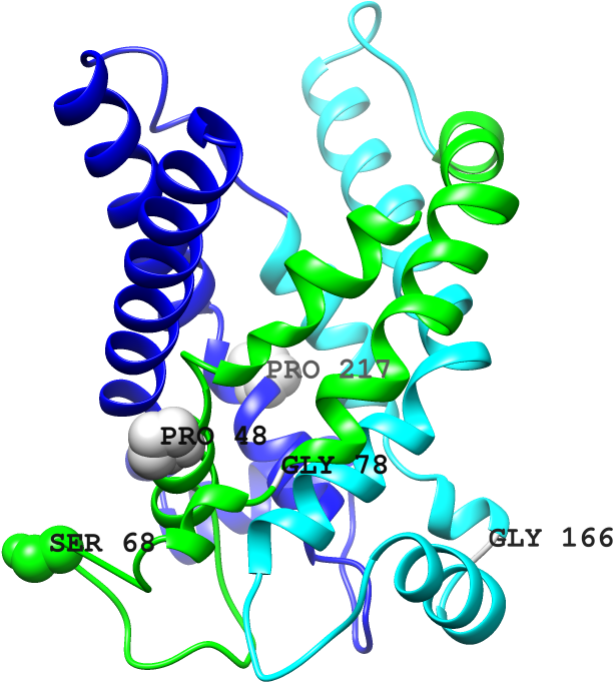
Three-dimensional structure model for Lpg1137. The three pseudorepeats indicated by color: green (1st), cyan (2nd) and dark blue (3rd). Serine 68 shown. Also, selected conserved residues shown: P48 (15), G78 (47), G166 (175), P217 (239), numbering in parentheses indicates positions in the sequence logo. Model obtained from the Phyre2 server using Protein Data Bank structure 4c9q (chain B) as template. Intermembrane space is on the top, mitochondrial matrix—on the bottom.

Identification of Lpg1137’s similarity to MCs allows the building of a model of its three-dimensional structure. Given the model, the special role of Ser68 can be addressed. In the structure model (see Fig. 3), this residue is located exposed in a loop region inside the mitochondrial matrix (or other organelle in whose membrane Lpg1137 might locate). Of note, S68 is not conserved among Lpg1137 close homologs, as would have been expected were it the catalytic residue (see Fig. 2). Thus, Ser68 might be mediating Lpg1137 interactions with other molecules or with Stx17 itself, which may be of importance for the downstream cleavage of Stx17.

Based on our data, it appears less likely that Lpg1137 is itself a serine protease, as advocated by Arasaki et al. This would signify a very unique evolutionary appearance of a catalytic function on a carrier-like protein. Such a scenario appears to be supported by one experiment (Arasaki et al., 2017) (see Fig. 3E in the Arasaki et al. paper) which is interpreted as showing protease activity of recombinant Lpg1137. The Western blot does not show the appearance of lower molecular mass species of the cleaved protein. Also, this result is shown without replication and is not quantitative.

The sequence motif G-L-S-G-G around Ser68 in Lpg1137 is described by Arasaki et al. as the occurrence of a generic motif G-X-S-X-[GA] which bears superficial similarity to the partial catalytic signature of a serine protease active site. However, the rest of the classic serine protease catalytic triad (Ser-His-Asp) is not mentioned by those authors nor was it detected by us (Arasaki et al., 2017). According to the Merops database, serine proteases can be grouped in 12 clans divided into 36 families (Rawlings, Barrett & Finn, 2016). In one of the best studied families, trypsin, the active site serine is located in a conserved motif, G-[DNE]-S-G-[GS]-[PAST]. PrositeScan analysis (Gattiker, Gasteiger & Bairoch, 2002) indicates that the generic motif G-X-S-X-[GA] is non-specific and is very often found in randomised sequence databases (1,496 matches on a sample of 5,000 Swiss-Prot shuffled sequences). Thus, a motif that occurs in every third random sequence is unlikely to be a sign of a functional site. The precise motif surrounding Ser68 in Lpg1137 (G-L-S-G-G) can be found in 1,235 sequences from the SwissProt database. However, only two of these are annotated as serine proteases. Therefore, the lack of recognizable His and Asp active site motifs and the poor specificity of the Ser68 motif make the similarity of Lpg1137 to known serine proteases doubtful.

**Figure 4:**
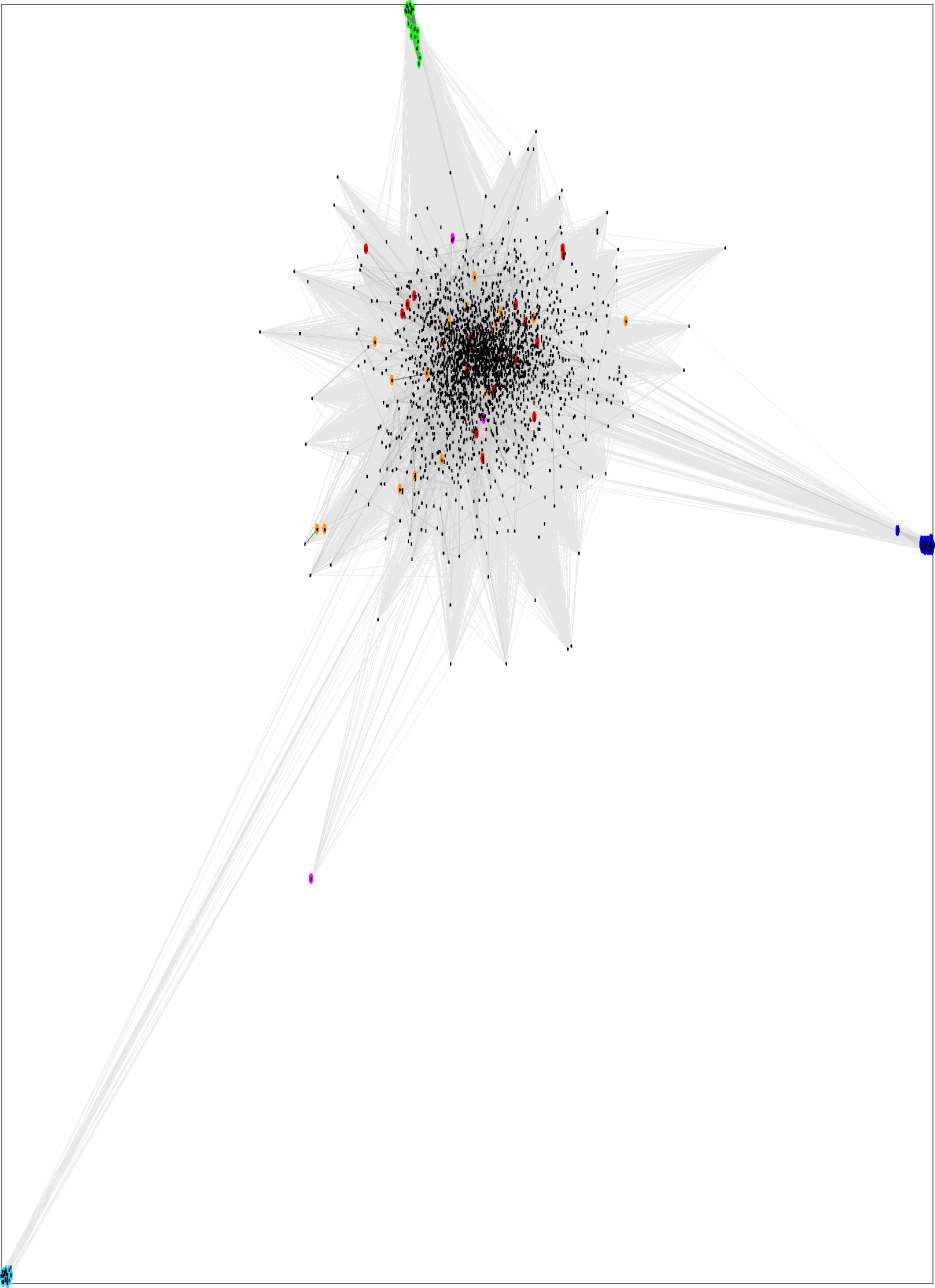
CLANS graph visualizing PSI-BLAST-detected sequence similarities between repeats of mitochondrial carrier proteins (Pfam: Mito_carr). The points represent sequences, and distances reflect the sequence dissimilarities. Black: eukaryotic repeats; red: viral repeats (Pfam); orange: bacterial repeats (Pfam); green: Lpg1137 homologues, 1st repeats; cyan: Lpg1137 homologues, 2nd repeats; dark blue: Lpg1137 homologues, 3rd repeats, magenta: *L. pneumophila* MC effector lncP (Dolezal et al., 2012).

When CLANS, the sequence similarity-based clustering algorithm, is applied to the set of Lpg1137 homologues and a representative set of MC repeats (see Fig. 4), it is obvious that the three sequence repeats of Lpg1137 are very distant from each other and from the rest of MC repeats. Indeed, in this analysis, all the eukaryotic MC repeats group in a single central cluster, while Lpg1137 repeats are located in distant outlier clusters.

Recently, another mitochondrial carrier *L. pneumophila* effector has been studied, lncP/LLO_1924 (Dolezal et al., 2012). This effector is somewhat less widespread in *Legionellas* than Lpg1137, and is found in 7 out of 41 genomes studied in the recent Burstein paper (Burstein et al., 2016). However, the lncP protein is only remotely similar to Lpg1137 (9% sequence identity in a FFAS03 sequence alignment with a significant Zscore equaling −41, see also Fig. 4). The role of lncP in infection is not clear; however, lncP can catalyze ATP efflux from mitochondria in infected cells (Dolezal et al., 2012).

Bacterial homologues of eukaryotic mitochondrial carriers are found in a handful of bacterial strains, usually infectious ones, including a few *Chlamydiales*, *Rickettsiales* and *Legionellales* (Pfam family Mito_carr, PF00153). These proteins have been hypothesized to be the results of horizontal gene transfer from eukaryotes and to be involved in infection (Dolezal et al., 2012). Lpg1137 is only a very distant homologue of those annotated bacterial MC proteins and the eukaryotic MC proteins (see Fig. 4) with the middle sequence repeat being most divergent.

## Conclusion

We present strong bioinformatic evidence that Lpg1137 is a mitochondrial carrier-like protein, a very distant homologue of SLC25 carriers. Nevertheless, current bioinformatic study does not constitute a proof that Lpg1137 is not a protease. What we present is strong evidence that Lpg1137 is not a homologue of known proteases, but rather a distant homologue of mitochondrial carriers. Building on the results of Arasaki et al., future experimental studies should include the solving of the three-dimensional structure of the protein and should cast light on its detailed function: be it proteolysis, small molecule transport across mitochondrial or other membrane, modulation of the activity of other MC proteins by oligomerization, or be it yet another role.

## Supplemental Information

10.7717/peerj.3849/supp-1 Supplemental Information 1 CLANS clustering file CLANS clustering file (MC pseudorepeats, as used for Fig. 4)

10.7717/peerj.3849/supp-2 Supplemental Information 2 PDB coordinate file, lpg1137 structure model PDB coordinate file, lpg1137 structure model (as used for Fig. 3)

## Additional Information and Declarations

### Data Availability

The following information was supplied regarding data availability: The raw data is supplied in the Supplemental Files.
